# Presence of anti-Müllerian hormone (AMH) during follicular development in the porcine ovary

**DOI:** 10.1371/journal.pone.0197894

**Published:** 2018-07-31

**Authors:** Fernanda R. C. L. Almeida, Natasja G. J. Costermans, Nicoline M. Soede, Annelies Bunschoten, Jaap Keijer, Bas Kemp, Katja J. Teerds

**Affiliations:** 1 Department of Morphology, Institute of Biological Sciences, Federal University of Minas Gerais, Av. Antônio Carlos, Belo Horizonte, Minas Gerais, Brazil; 2 Adaptation Physiology Group, Department of Animal Sciences, Wageningen University, Wageningen, Netherlands; 3 Human and Animal Physiology, Department of Animal Sciences, Wageningen University, Wageningen, Netherlands; INIA, SPAIN

## Abstract

**Background:**

Anti-Müllerian hormone (AMH) is expressed by granulosa cells of developing follicles and plays an inhibiting role in the cyclic process of follicular recruitment by determining follicle-stimulating hormone threshold levels. Knowledge of AMH expression in the porcine ovary is important to understand the reproductive efficiency in female pigs.

**Research aim:**

In the present study we investigated the expression of AMH during follicular development in prepubertal and adult female pigs by immunohistochemistry, laser capture micro-dissection and RT-qPCR.

**Results and conclusion:**

Although in many aspects the immunohistochemical localization of AMH in the porcine ovary does not differ from other species, there are also some striking differences. As in most species, AMH appears for the first time during porcine follicular development in the fusiform granulosa cells of recruited primordial follicles and continues to be present in granulosa cells up to the antral stage. By the time follicles reach the pre-ovulatory stage, AMH staining intensity increases significantly, and both protein and gene expression is not restricted to granulosa cells; theca cells now also express AMH. AMH continues to be expressed after ovulation in the luteal cells of the corpus luteum, a phenomenon unique to the porcine ovary. The physiological function of AMH in the corpus luteum is at present not clear. One can speculate that it may contribute to the regulation of the cyclic recruitment of small antral follicles. By avoiding premature exhaustion of the ovarian follicular reserve, AMH may contribute to optimization of reproductive performance in female pigs.

## Introduction

Anti-Müllerian hormone (AMH) is a dimeric protein and a member of the transforming growth factor B (TGFB) superfamily of glycoproteins. AMH was initially identified as a product of Sertoli cells in the foetal testis where it induces regression of the Müllerian ducts at the time of testicular differentiation [1, 2]. In females, in contrast to males, AMH is not expressed during sex differentiation, but appears for the first time in the neonatal ovary in granulosa cells of recruited primordial follicles [3]. AMH continues to be expressed in granulosa cells of growing follicles until these reach the stage of cyclic recruitment, a process controlled by the pituitary gonadotropin follicle-stimulating hormone (FSH) [4–7]. In the mouse ovary, this selection occurs at the early antral stage [3], in the human ovary in antral follicles of 4–6 mm in size [8].

Studies in AMH null mice revealed that AMH is involved in the regulation of the rate of initial recruitment of primordial follicles into the growing pool. In these knockout mice, more primordial follicles are recruited, and as a consequence, the primordial follicle pool rapidly decreases in size [9–11]. In line with these results, exposure of neonatal mouse ovaries or human ovarian tissue biopsies to elevated levels of AMH leads to a significant reduction in the number of growing follicles [12,13]. These data suggest that AMH plays an inhibitory role in the recruitment of primordial follicles [12,14], thus avoiding premature exhaustion of the ovarian follicular reserve [15].

Studies in AMH null mice further suggest that AMH modulates the sensitivity of small growing follicles to FSH [9]. Evidence for this hypothesis comes from *in vitro* studies, which revealed that AMH inhibits FSH stimulated growth of small follicles [3]. The close interdependent relation between AMH and FSH activity is further strengthened by the inverse correlation between AMH follicular fluid concentration and FSH stimulated oestradiol concentration in small antral follicles [16]. This negative correlation is a consequence of the AMH induced repression of the expression of aromatase (*Cyp19a1*) [17–20]. These observations suggest that AMH is not only involved in the initial recruitment of primordial follicles, but also plays a role in determining FSH threshold levels for cyclic recruitment of growing antral follicles.

A representative marker to establish the quantitative aspects of the ovarian follicular reserve, which can be easily measured in blood, is of great value to determine reproductive capacity [21]. Indeed, it is shown in mice, humans, horses, sheep and cattle that serum AMH levels strongly correlate with the number of growing follicles in the ovary [14, 22–28]. As the size of the primordial follicle pool reflects the number of growing follicles, AMH concentration in plasma and in follicular fluid is considered to be a marker to assess the quantitative aspects of the ovarian follicular reserve [23, 29, 30]. This concept has renewed the interest in AMH in ruminants and other domestic species. The recent availability of sensitive and multiple species AMH immunoassays has made it possible to accurately measure AMH in blood and follicular fluid of these species (reviewed in [15]).

Expression of AMH has been investigated in a considerable number of species such as rodents [12], sheep [31], cattle [7], horses [24], dogs [32] and humans (reviewed in [11]). However, there is hardly any information available concerning AMH expression in the adult porcine ovary. In one of the few available studies Monniaux et al. [15] report that there is no significant difference in AMH concentration in follicular fluid of small- (1–3 mm) and large-sized (>5 mm) porcine antral follicles.

In the pig industry, ovulation rate is an important component of reproductive efficiency [33]. As AMH is not only involved in initial follicle recruitment but also seems to play a role in determining FSH threshold levels for cyclic recruitment of growing antral follicles, knowledge of AMH expression in the porcine ovary will contribute to the optimization of reproductive efficiency in female pigs. In the present research, we therefore investigated the localization of AMH in the porcine ovary during follicular development in prepubertal and adult pigs by immunohistochemistry, laser capture microdissection and qPCR. We show that in the adult sow, in contrast to previous reports in numerous different species, AMH is not only present in granulosa cells of growing follicles but also in theca cells of preovulatory follicles and corpora lutea, implicating a possible involvement of AMH in regulating pig reproductive capacity.

## Material and methods

### Animals

For immunohistochemical purposes, ovaries were collected immediately after slaughter from: i) 12 prepubertal gilts (TOPIGS 20, crossbred Dutch landrace and Yorkshire) with an average age of 75 to 85 days, ii) five cyclic adult sows (TOPIGS 20), and iii) two pregnant sows (TOPIGS 20) at approximately day 30 of pregnancy. The ovaries of the cyclic sows were studied macroscopically to estimate the phase of the oestrus cycle. When the ovaries contained follicles of pre-ovulatory size (7–9 mm) the sow was classified as being in the late follicular phase (n = 4) [34]. One ovary was in the luteal phase of the oestrus cycle as was determined by macroscopically visible corpora lutea on the ovary.

After collection, the right ovaries from all animals were immediately fixed in 4% phosphate buffered paraformaldehyde at 4°C for 24 hours, followed by embedding in paraffin. Five μm thick paraffin sections were cut and mounted on super frost plus slides (Menzel, Braunschweig, Germany). Buffered paraformaldehyde fixed adult rat ovaries were used as positive control tissue. This tissue was treated in exactly the same way as the porcine ovarian tissue.

The experiment in which we used ovaries from prepubertal gilts, was approved by the Ethics Committee for Animal Experimentation of Wageningen University (DEC2010130). The ovaries from the adult cyclic and pregnant sows were obtained from the slaughterhouse.

For gene expression analysis, ovaries of three cyclic adult sows were collected from the slaughterhouse; one sow in the early follicular phase, one sow in the late follicular phase and one sow in the luteal phase of the oestrus cycle. After collection, the ovaries were immediately fixed in modified methacarn (methanol and glacial acetic acid, 8:1) at room temperature for 24 hours on a rocking table. After fixation, the ovaries were cut in pieces of 1 by 1 cm and embedded in paraffin. Five μm thick paraffin sections were cut and mounted on superfrost plus slides (Menzel).

### Immunohistochemistry

To determine the presence of AMH in porcine ovaries, immunohistochemistry was performed according to Durlinger et al. [12] with minor modifications. Staining of all slides was carried out simultaneously in a single session. Briefly, sections were dewaxed, rehydrated and microwaved for 3 X 5 min in 0.1 M sodium citrate buffer (pH 6) for epitope antigen retrieval, cooled down to room temperature and rinsed with phosphate buffered saline pH7.4 (PBS). Endogenous peroxidase activity was blocked with 3% (v/v) hydrogen peroxide in methanol solution for 30 min. Sections were pre-incubated with 5% (wt/v) normal rabbit serum in PBS for 30 min at room temperature. Subsequently, the sections were incubated overnight at 4°C in a humid chamber with the primary polyclonal goat anti-MIS/AMH antibody (sc-6886, lot# H2208, Santa Cruz Biotechnology Inc, Santa Cruz, CA, USA) diluted 1:100 (v/v) in PBS + 0.05% BSA-c (Aurion, Wageningen, The Netherlands). Next, the sections were treated with a secondary biotin labelled rabbit-anti-goat antibody (Vector Laboratories, Burlingame, CA, USA) diluted 1:200 (v/v) in PBS-BSAc for 1 h at room temperature. This was followed by incubation with avidin-biotin complex (ABC, diluted 1:500 (v/v) in PBS-BSAc (Vector stain kit Elite, Vector Laboratories) for 60 min at room temperature. Bound antibody was visualized using the Immpact DAB kit (stock solution diluted 1:200 (v/v); Vector Laboratories). Sections were counterstained with Mayer’s haematoxylin. In control sections, either the primary antibody was replaced by PBS/BSAc (data not shown), or sections were incubated with normal goat serum or 5% (wt/v) BSA/PBS in the absence of the primary antibody. In the first case, no brown staining was observed (data not shown), while in the latter two cases some non-specific staining in the oocyte nucleus and follicular fluid was sometimes observed. Ovaries from adult rats were used as a positive control. The immunohistochemical staining procedures were repeated at least five times for all animals tested.

### Follicular nomenclature

Follicles were classified according to Flaws [35] with modifications. Briefly, follicles were identified as healthy if they contained an intact oocyte, organized granulosa and theca layers, and no pyknotic nuclei. Atretic follicles either contained a degenerating oocyte, disorganized granulosa cells, pyknotic nuclei, shrunken granulosa cells or apoptotic bodies. Follicles were classified as resting primordial if they contained an intact oocyte with a visible nucleus surrounded by a single layer of flat-shaped follicle epithelial cells. Follicles in which both flat-shaped follicle epithelial cells and some cuboidal granulosa cells surround the oocyte were classified as recruited primordial follicles. Follicles were scored as primary if they consisted of an intact, enlarged oocyte surrounded by a single layer of mainly cuboidal granulosa cells. Follicles were scored as preantral if they contained an oocyte and more than one layer of granulosa cells and a developing theca cell layer. Follicles in which at least one antral cavity was present with a diameter larger than the oocyte diameter, and in which the oocyte was still located more or less in the centre of the follicle, were scored as early antral. In large antral follicles, the oocyte was located a-centrally surrounded by cumulus granulosa cells and a large antral cavity. The theca cell layer surrounding the granulosa did not show signs of disintegration of the basal membrane between the granulosa and theca layer. In preovulatory follicles, extensive folding of the theca and granulosa cell layers was observed due to disintegration of the basal membrane separating granulosa and theca layer.

### Semi-quantitative analysis of AMH staining intensity

Quantification of the AMH staining intensity was performed by digital morphometric analyses. Thirty frames of 53,333.4 μm^2^ from each ovary were randomly digitalized using a JVCTK1270 micro-camera and at least 10 well preserved follicles from each class were counted with the use of KS300 software coupled to a Carl Zeiss image analyser (Carl Zeiss, Oberkochen, Germany). The optical density (OD) calculation method was used according to Costa et al. [36], and was performed as follows:

- Deactivation of the red-green-blue (RGB) signal and the automatic gain control of the micro-camera to allow black and white capture through the KS300 software;
- OD measurement with the use of a GG495 interference filter (Schott, Mainz, Germany) at a wavelength of 500 nm, which was absorbed only by the DAB solution;
- Images of lightly counterstained sections were captured with a ×40 objective;
- Measurement of objects only larger than 30 pixels;
- Calibration of the condenser aperture during Köhler illumination, so that the geometric calibration was done separately in the vertical and horizontal axes through a micrometric slide (Carl Zeiss).

The algorithm function was used to select the positive areas (granulosa cells) and for creation of a binary image. Oocytes were excluded from the analysis, as the staining in the oocyte cytoplasm was nonspecific background staining. OD was obtained using the ratio between the transmitted light and the incident light at 500 nm wavelength, according to Beer's law:

$$OD=‑\log\;I/I_{0}.,$$

Where I0 is the total intensity of light trans-illuminating areas not marked in the material and I is the intensity of light transmitted from any given pixel of the analyzed region. In a digital specimen image, I is proportional to pixel grey value. Since each labeled granulosa cell is composed of many pixels, the sum of grey value (SUMD) divided by the area (in pixels) gives the I value, which is equal to MEAND (densitometry mean). As the brightness values range from 0 to 255 (8 bit range) in the image analyzer utilized, the theoretical I_0_ is equal to 256. Therefore, I0 represents the background tissue mean pixel values, which is obtained through the generation of a binary image from this region and subsequent calculation of MEAND. The inclusion of the background grey value in the OD calculation formula was done to normalize the data. The background grey value of the present material was equal to 141, thus the formula for OD calculation was: OD = -log MEAND/141.

OD values, expressed as grey units, close to zero correspond to strong immunolabeling and, consequently, more antigen bound by the AMH antibody.

### Laser capture microdissection

To determine AMH gene expression in granulosa (luteal) cells and theca (luteal) cells, first immunostaining was performed on methacarn fixed tissue according to the methods described above to identify the AMH-positive cells. The subsequent sections were stained with Mayer’s haematoxylin and used to perform laser capture micro-dissection (LCM), according to DeCarlo et al. [37] with minor modifications. Briefly, after staining, sections were dehydrated and air-dried for 5 minutes. AMH positive granulosa cells of an antral follicle from an ovary in the early folicular phase and theca cells of a pre-ovulatory follicle from an ovary in the late follicular phase as well as corpora luteal cells from the ovary in the luteal phase of the oestrus cycle were micro-dissected under 40x magnification (Palm Laser Dissection Microscope, Zeiss, Sliedrecht, the Netherlands). Around 1x10^4^ granulosa, theca or corpora lutea cells were collected into silicon coated Adhesive cap500 caps (Zeiss, Gottingen, Germany), respectively and analysed separately. After microdissection, the cells in the caps were lysed with 20 μl of extraction buffer (Picopure RNA Isolation kit, Arcturus, San Diego, CA, USA) and incubated for 30 min at 42°C. The resulting cell lysates were stored at -80°C until RNA isolation.

### RNA isolation, cDNA synthesis and pre-amplification

Total RNA from the cell lysates was extracted using the Picopure RNA Isolation kit (Arcturus, San Diego, CA, USA). In addition, RNA from pig liver was isolated using the RNeasy mini kit (Qiagen, Venlo, The Netherlands), to serve as a negative control. In both protocols, on-column DNAse treatment (Qiagen) was used. RNA concentration and purity was measured with the Nanodrop spectrophotometer (IsoGen Life Sciences, Maarsen, The Netherlands). Approximately 30–40 ng RNA was reversely transcribed using the iScript cDNA synthesis kit (Bio-Rad, Veenendaal, The Netherlands). cDNA of each sample was pre-amplified for 22 cycles using Sso Advanced SYBR Green (Bio-Rad). RNA isolation, cDNA synthesis and pre-amplification were performed according to manufacturers’ instructions. A primer pair for amplification of the AMH gene was designed using the NCBI primer design tool and the sequences used were: (forward) ‘5-AAGCTCCTCATCAGCCTGTCT-3’ and (reverse) ‘5- ATTGGGGCGATCGGGTTTG-3’. The PCR product size of the AMH gene was 145 bp.

### AMH gene expression by RT-qPCR

AMH gene expression in granulosa and theca cells, corpora lutea and liver (negative control) was analysed by qPCR using iQ SYBR Green Supermix (Bio-Rad) and the CFX96 Real-time system (Bio-Rad). The cDNA was denatured for 3 minutes at 95°C, followed by 40 two-step amplification cycles (15 s at 95°C, 45 s at 64°C).

Data were collected using Bio-Rad CFX manager. To test the quality of the PCR products, a water control and a RNA sample without reverse transcriptase during cDNA synthesis were taken along. As an additional quality check, a standard curve was made using serial dilutions of a pool prepared from all pre-amplified cDNA samples (criteria: ≤ 90% efficiency ≥ 110% and correlation coefficiency ≥ 0.99). The efficiency of the AMH primer pair was 97.8% and the correlation coefficient was 0.996. DNA gel electrophoresis (2% agarose gel in 1x TAE buffer) was performed using the GeneRuler Ultra Low Range DNA ladder (Fisher Scientific, Landsmeer, The Netherlands) to determine the size of the PCR products. After staining with SYBR Safe DNA gel stain (Invitrogen, Carlsbad, CA), fragments were visualised under UV light.

### Statistical analysis

Optical density values were tested for normality prior to analyses, using the univariate procedure of the Statistical Analysis System (SAS Institute, Cary, NC, USA, version 8.2). As these values did not present a normal distribution, the optical density was log-transformed prior to analysis. Data were analysed as a randomized design and the statistical model included female pigs as fixed factor and follicle class as random factor. Treatment effects on optical density were analysed using the general linear model (GLM) procedure of SAS. In the event that significant treatment effects were established, multiple comparisons were performed using probability of differences (pdiff) between least square means, adjusted by Tukey-Kramer with P < 0.05 being considered significant.

## Results

### Immunohistochemistry

**Fig 1:**
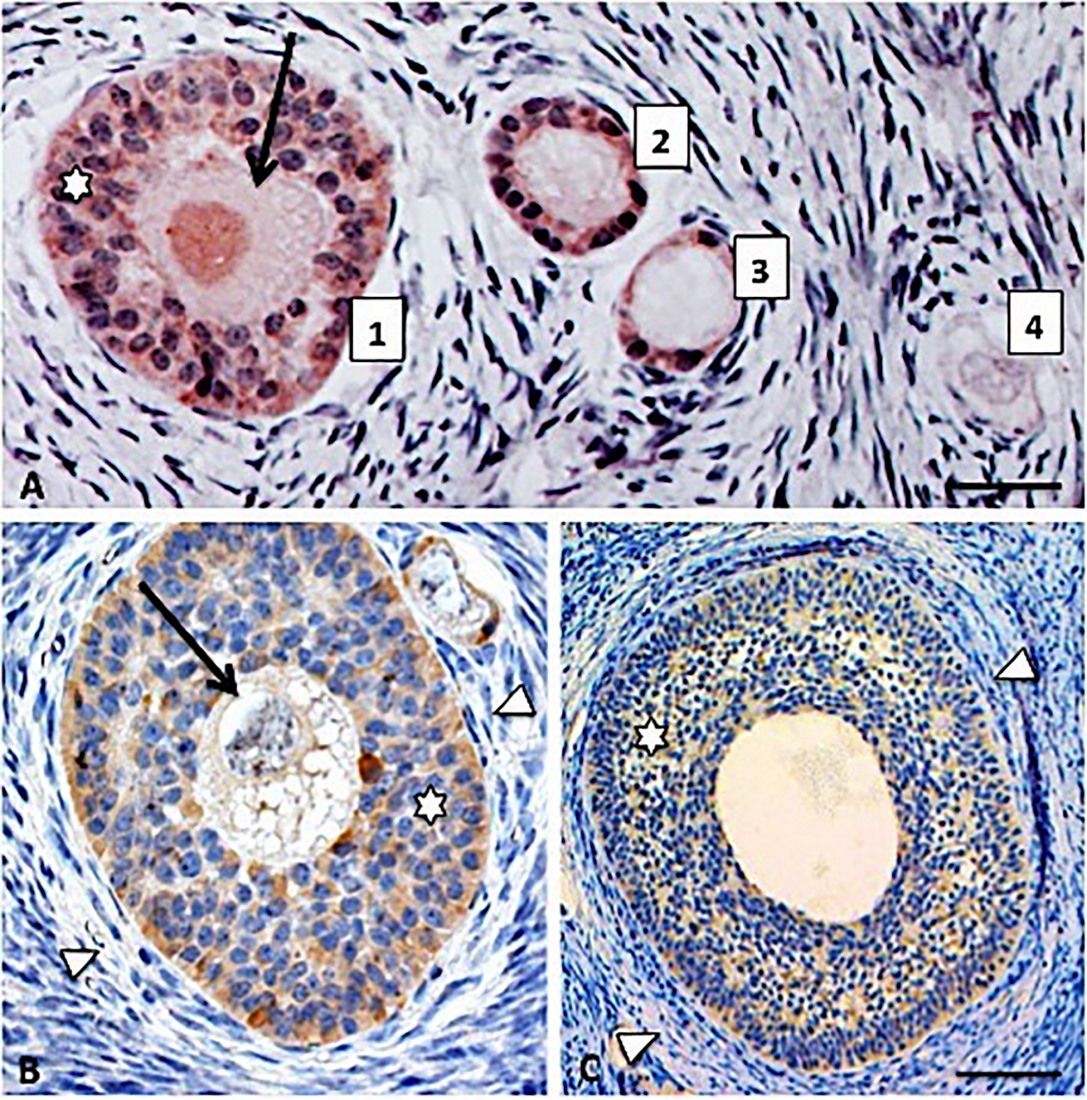
Representative AMH staining (brown) in the porcine ovary: (A) 1. Small preantral follicle, 2. Primary follicle, 3. Recruited primordial follicle, 4. Quiescent primordial follicle in which AMH staining is absent; (B) Large preantral follicle; (C) Small antral follicle. Oocytes are indicated by arrows, AMH positive granulosa cells by asterisks and theca cells by arrowheads. Scale bars represent 15 μm (A), 30 μm (B), 60 μm (C).

**Fig 2:**
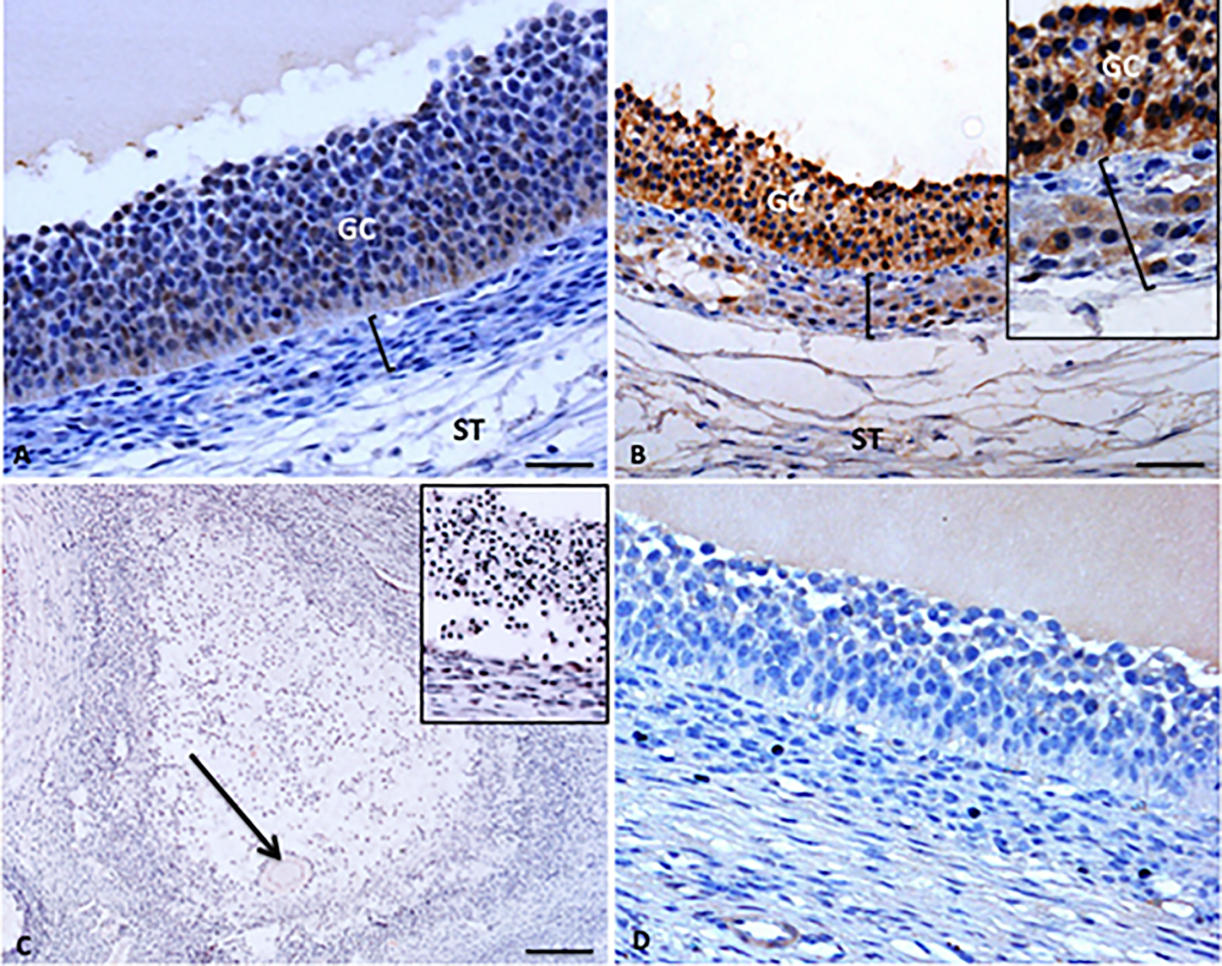
Semi-quantitative analysis of AMH staining during follicular development in the porcine ovary. AMH staining was quantified in recruited primordial, primary, small preantral, large preantral, small antral, large antral and preovulatory follicles using KS300 software coupled to a Zeiss image analyzer. Low OD values correspond to strong AMH immunostaining. OD values are expressed as grey units (for more details see Materials and Methods). Values are expressed as mean ± SEM. AMH negative stroma tissue served as a control. Different characters represent significant differences in AMH staining intensity between follicle types (b–P < 0.05; c–P < 0.01).

No differences in AMH staining intensity were observed between ovaries of the 19 prepubertal and five adult non-pregnant sows and therefore these two groups of animals were combined for immunohistochemical evaluation of the presence of AMH during follicular development. In quiescent primordial follicles AMH staining was absent. However, when primordial follicles were recruited into the pool of growing follicles, as shown by the presence of some cuboidal granulosa cells next to flat-shaped epithelial cells, AMH staining could be detected in the cells surrounding the oocyte (Fig 1A, Panel A in S1 Fig). Clear AMH immunostaining was observed in the granulosa cells of primary, small and large preantral follicles and in early antral follicles at the start of antrum formation when follicular fluid accumulated between the granulosa cells (Fig 1, Panel A in S1 Fig). This observation was supported by semi-quantitative analysis of AMH staining during follicular development (Fig 2). This staining pattern was comparable to what is observed in the rat ovary (S2 Fig; [6]).

**Fig 3:**
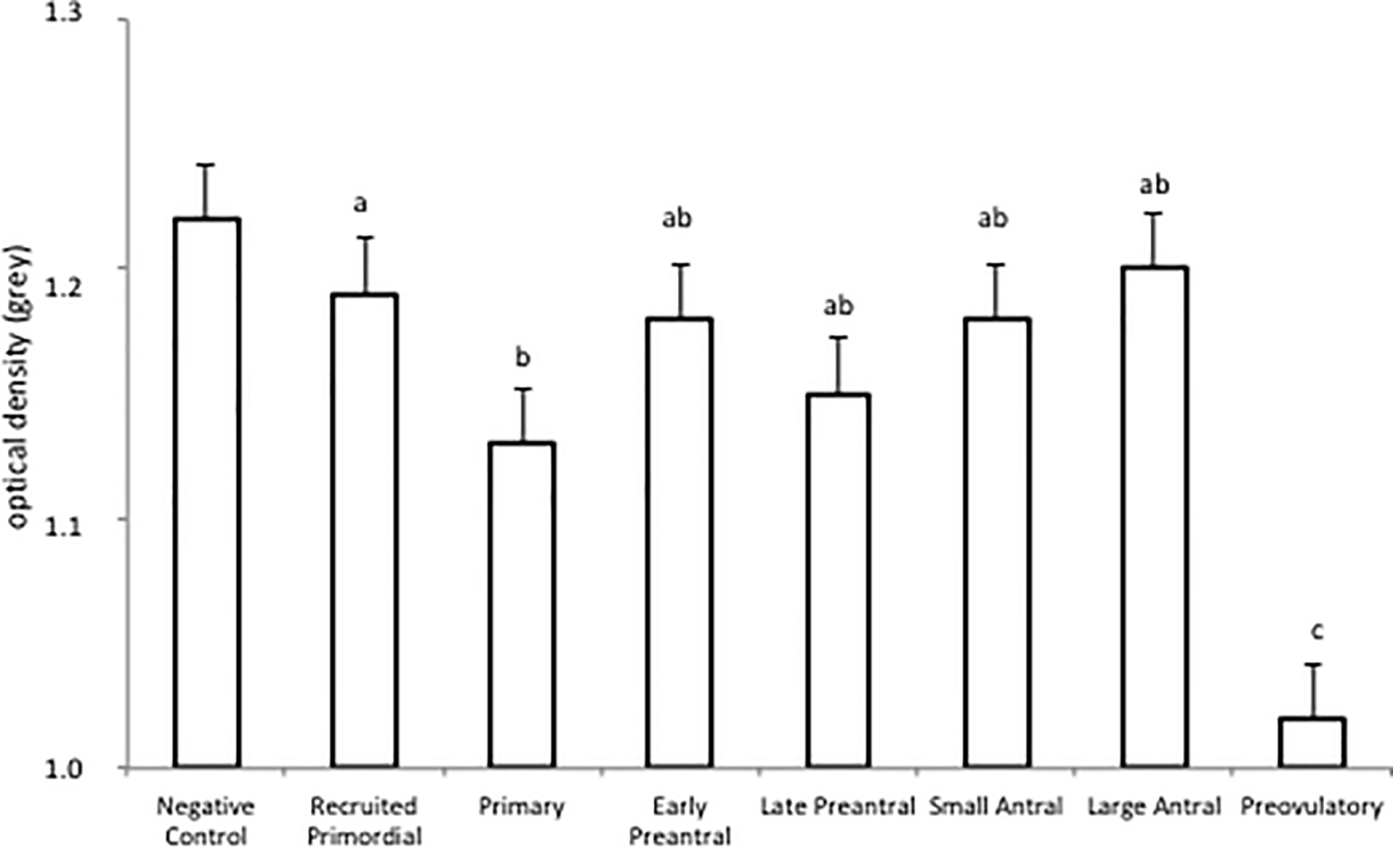
Representative AMH staining (brown) in adult sow ovary. (A) Moderate to strong heterogeneous AMH staining is observed in granulosa cells of a large antral follicle; (B) In preovulatory follicles, strong AMH staining is present in the granulosa cells, while moderate to strong heterogeneous staining is found in the surrounding theca cells–insert detail of AMH positive theca cells; (C) In atretic follicles, AMH staining is faint to absent–insert detail of follicular wall of an atretic follicle; (D) In control incubations in which the primary antibody is replaced by normal goat serum some faint background staining is present in the follicular fluid. GC—granulosa layer; black bar—theca layer; black arrow—oocyte; ST—stroma. Scale bars represent 30μm (A,D), 45 μm, (B), 80 μm (C).

In large antral follicles, AMH staining in the granulosa cells was somewhat heterogeneous and therefore gave the impression to be somewhat less intense compared to other stages of follicular development, though this did not lead to significant differences in semi-quantitative staining intensity (Figs 2 and 3A). In four animals in the late follicular phase of the oestrus cycle strong AMH staining was observed in preovulatory follicles both in the granulosa and theca cells, resulting in a significant increase in semi-quantitative staining intensity (Figs 2 and 3B).

**Fig 4:**
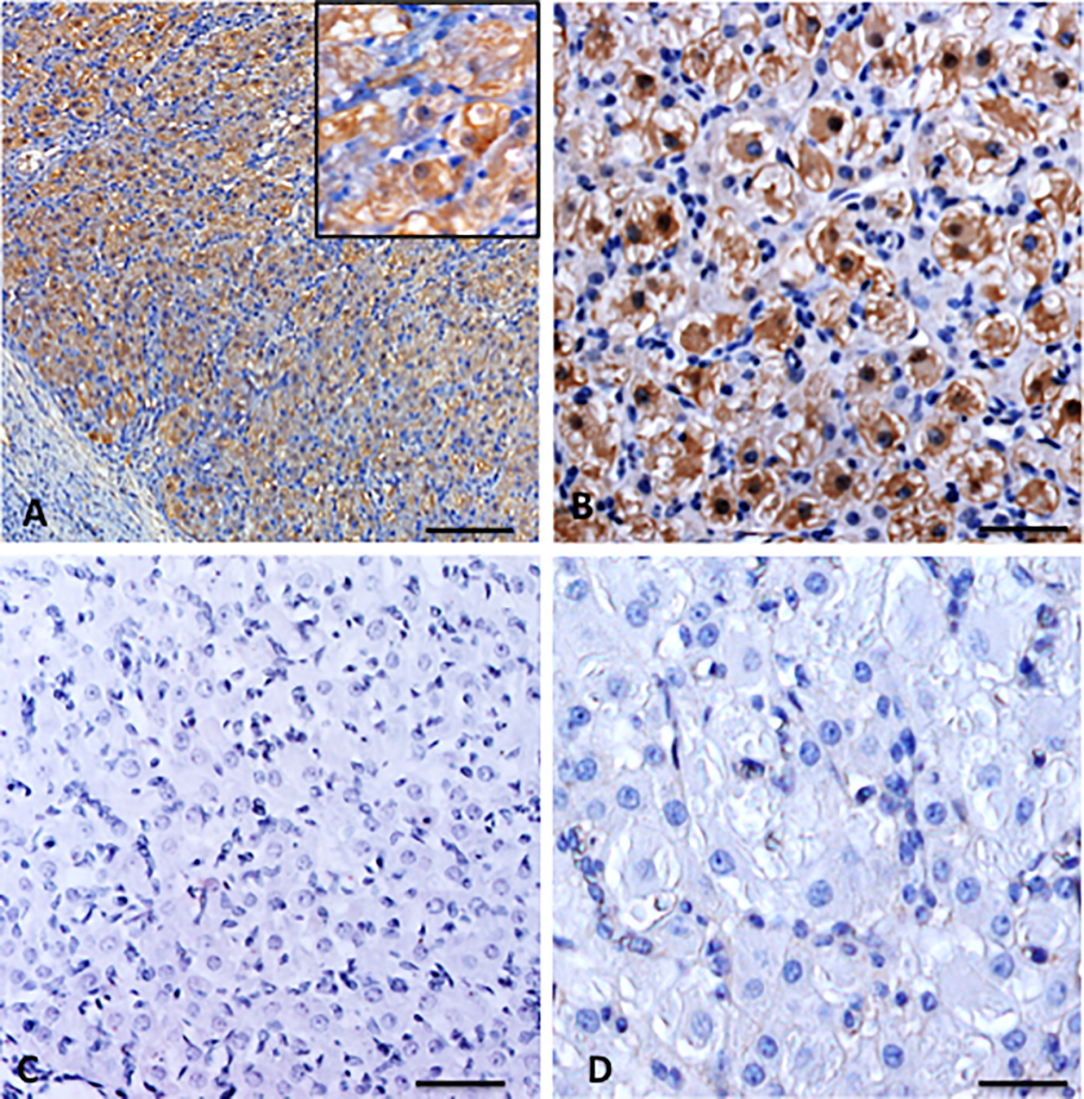
Representative AMH staining (brown) in corpora lutea. (A) Sow ovary, clear staining in the luteal cells–insert higher magnification of AMH positive luteal cells; (B) AMH positive luteal cells is a corpus luteum of a 30-day pregnant sow; (C) Rat ovary, note the absence of AMH staining in the luteal cells; (D) Sow ovary, control incubation in which the primary antibody was replaced by normal goat serum. No staining can be detected in the luteal cells under these conditions. Scale bars represent 80 μm (A), 30 μm (B,C), 20 μm (D).

In one animal in the luteal phase of the oestrus cycle persistent AMH staining was observed in the granulosa and thecal luteal cells in the corpora lutea. This is in contrast to what has been reported for the rat ovary (Fig 4A, Panel B in S2 Fig; [6]). In case of pregnancy when the corpora lutea did not undergo regression, the luteal cells continued to stain positively for AMH, at least up to approximately day 30 of pregnancy (Fig 4B).

In atretic antral follicles, AMH staining was faint to absent in both granulosa and theca cells (Fig 3C).

### Gene expression

**Fig 5:**
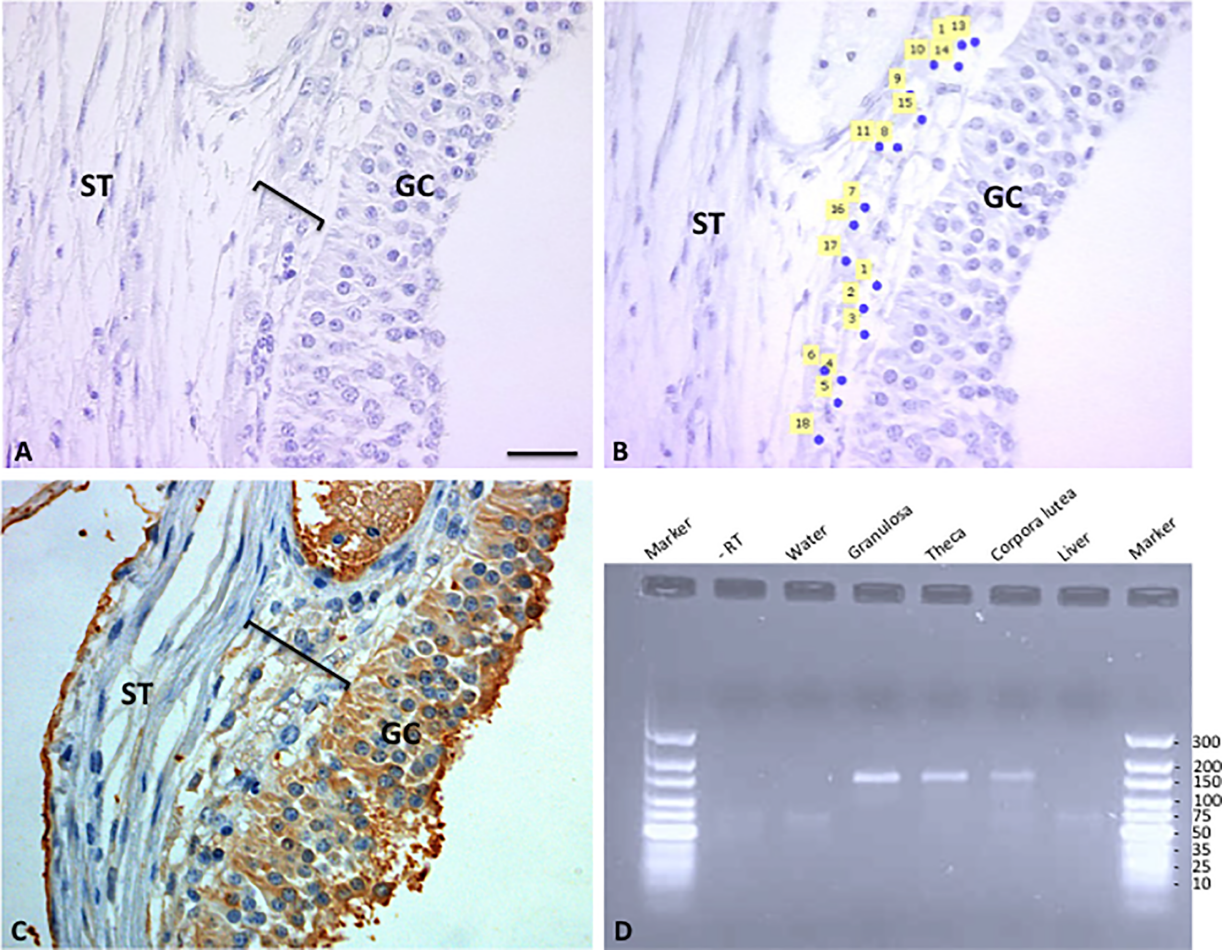
Representative pictures used for LCM capturing of theca cells in preovulatory follicles. (A) Preovualtory follicle prior to capturing of cells; (B) Same follicle after laser capturing of cells, areas where cells have been captured are indicated by numbered yellow squares; (C) Same follicle stained with the AMH antibody, positive granulosa and theca cells are indicated (brown staining); (D) Agarose gel electrophoresis of AMH cDNA products after RT-PCR (product size—145 bp), lane 1 marker, lane 2—RT (control), lane 3 water (control), lane 4 granulosa cells, lane 5 theca cells, lane 6 luteal cells, lane 7 liver (negative control), lane 8 marker. GC—granulosa layer; black bar—theca layer; ST—stroma. Scale bar represents 30μm (A-C).

Methacarn fixed ovarian tissue sections were first stained with the antibody against AMH to demonstrate the presence of AMH protein in the granulosa and thecal cells prior to the capturing of cells (Fig 5A). In order to determine whether the different AMH-immunopositive cell-types also express AMH mRNA, successive sections were used to isolate RNA from by laser capture micro-dissection (Fig 5B). AMH gene expression was detected by qPCR in granulosa cells from follicles in the early follicular phase of the oestrus cycle, in theca cells from preovulatory follicles and luteal cells (Fig 5C). As expected, the liver sample, which was used as a negative control, did not express AMH mRNA (Fig 5D).

## Discussion

The present study describes the presence of AMH in the porcine ovary during follicular development. Despite in many aspects the immunolocalization of AMH during follicular development in the porcine ovary is comparable to other species, there are also some striking differences (see Table 1 for an overview of ovarian AMH distribution among different species). Immunohistochemical labelling and in situ hybridization studies have shown that 4AMH is found in the (pre)granulosa cells, not in the oocyte or theca cells, except some diffuse immunostaining in theca cells in the human (38) and in the caprine ovary (39) (Table 1). In the porcine ovary however AMH immunostaining is not restricted to the granulosa cells, as in preovulatory follicles the theca cells surrounding the granulosa layer also express AMH immunostaining. This expression persists following ovulation in the luteal cells, a phenomenon unique for the porcine ovary, and which may have implications for a possible function of AMH during follicular development in the pig.

**Table 1:** Comparative analysis of the presence of AMH in the female ovary.

Species	Primordial follicles	Primary follicles	Preantral follicles	Small antral follicles	Large antral follicles	Preovulatory follicles	Atretic follicles	Corpus luteum	References
Porcine	- to +	++	++	++	+ to ++	++	-	++	this study
Human	-/+ to +	+	++	++	+ to ++	-/+	-/+	-	8, 39, 49, 51
Bonnet monkey	-/+	+	++	++	+	-/+	-/+	-	49
Squirrel monkey	-	+	+	ND	ND	ND	ND	ND	52
Baboon	+	++	+	-/+	ND	ND	ND	ND	53
Rhesus Macauqe	-	-/+ to +	-/+ to ++	-/+ to ++	ND	ND	ND	ND	54
Marmoset	-	+	++	++	+	ND	-/+	ND	55
Mouse	-	+	+/++	+/++	-/+	-	-/-/+	-	23, 56, 57
Rat	-	+	++	++	-/+	-	-/+ to +	- to -/+	5, 58, this study
Siberian hamster	ND	++	++	+	+	ND	ND	-	58, 60
Brushtail possum	-	+	++	++	++	-	ND	-	61
Tamar wallaby	-	-/+	+/++	++	++	ND	-/+	ND	62
Ovine	-	-/+	+/++	++	+	+	ND	-	31, 63, 64
Caprine	-/+	++	-/+/++	-/+/++	-/+	-/+	ND	ND	40, 47
Bovine	-	- to +	+ to ++	++	+	ND	-/+	-	65, 66
Equine	-	-/+	++	++	ND	ND	-	ND	24, 50
Hen^a^	ND	ND	++	++	-/+ to +	ND	ND	ND	67
Teleost fish^b^	ND	ND	++	++	ND	ND	ND	ND	68, 69

^a^In the hen, strong AMH staining is found in small follicles from 150 μm to 6 mm, stages comparable to preantral and early antral follicles in mammals. In 6–12 mm follicles, stages comparable to (large) antral follicles AMH staining decreases rapidly.

^b^In teleost fish, AMH is found in growing follicles in the oocytes and the follicular layer surrounding the oocytes, stages comparable to preantral and small antral follicles in mammals.

Abbreviations:—no staining; -/+ faint to absent staining; + moderate staining; ++ strong staining; ND not determined

The relatively stable immunohistochemical-labelling pattern during antral follicular development in the porcine ovary offers support to preliminary data by Monniaux et al. [15]. These authors reported that in contrast to the bovine and caprine ovary in which AMH levels in growing follicles gradually decrease, in the porcine ovary AMH levels in follicular fluid of small, medium and large antral follicles did not differ significantly from each other. Although these observations are interesting one has to keep in mind that AMH levels in porcine follicular fluid are around 300 to 500-fold lower compared to what has been measured in cows, sheep and goats [15]. Together with the observations of the present study, it may therefore be questionable whether in pigs granulosa cell-derived AMH plays a significant role in the modulation of FSH sensitivity of growing follicles, as has been postulated for rodents [3, 6, 38].

The appearance of intense AMH immunostaining in both granulosa and theca cells of preovulatory follicles is somewhat surprising as reports on the presence of AMH in theca cells are limited. Stubbs et al. [39] were the first to report the presence of AMH in theca cells and surrounding stroma cells in a few growing large antral follicles in the human ovary. The study of Rocha et al. [40] shows a diffuse AMH staining in granulosa and theca cells of small antral follicles in the caprine ovary. These authors have not investigated whether AMH staining continues to be present in theca cells up to the preovulatory stage.

Observations by Monniaux [15] suggest it is very well possible that in the caprine ovary AMH expression in granulosa and theca cells becomes less with increasing antral follicle size, as the AMH concentration in follicular fluid of antral follicles decreases with increasing follicle size. Whether theca cells are capable of AMH synthesis was up to now not clear. Stubbs et al. [39], hypothesized that the AMH staining in theca cells in the human ovary was probably due to diffusion from the granulosa cells. The present study is the first to show that this is not the case in the pig ovary, as we demonstrate by qPCR that theca cells of preovulatory follicles express the AMH gene and protein. The pig is the only species thus far in which AMH expression continues to be present in preovulatory follicles and corpora lutea. Diffuse AMH staining in the stroma surrounding the preovulatory follicles, as reported in the human ovary [38], and corpora lutea is negligible in the pig. We did not further quantify AMH gene expression in theca and luteal cells as this was beyond the aim of the present study.

The persistent presence of AMH after ovulation in the corpus luteum may be of physiological importance. In contrast to other species in which progesterone levels become elevated around the time of the LH surge, in the pig it takes between 40 to 48 h after the LH surge before progesterone levels start to increase [41]. Consequently, FSH levels continue to increase after the LH surge in the pig, reaching maximal levels approximately 3 days following the LH surge after which they gradually decrease [42]. FSH is an essential factor in the stimulation of preantral and antral follicular growth. In the absence of FSH, follicles do not develop beyond the large preantral stage [43, 44]; reduced levels of FSH induce atresia of growing follicles. Due to these persistently high FSH levels after the LH surge, small antral follicles in the porcine ovary may be challenged during the early lutea phase to enter the pool of growing follicles. We hypothesize that the consistent production of AMH in luteal cells together with granulosa cell AMH production will result in the continuous presence of AMH in the circulation and follicular fluid during the early luteal phase, which may influence follicular sensitivity to FSH thereby preventing premature recruitment of small antral follicles into the growing pool. Another explanation for the presence of AMH in luteal cells may be that it exhibits a role in the local regulation of progesterone production in the corpus luteum. This assumption is supported by the study of Prapa et al. [45], who reported that AMH exposure of luteinized human granulosa cells in vitro, decreased basal progesterone production. AMH is a member of the transforming growth factor B (TGFB) superfamily, that further includes bone morphogenetic proteins (BMPs), activins, inhibins and growth and differentiation factor 9 (GDF9). BMPs, in particular BMP6, have been reported to suppress bovine granulosa and thecal lutein cell progesterone production in vitro [46], while BMP15 has been shown to suppress progesterone production in human granulosa lutein cells [45]. These observations emphasize that it may be worthwhile to investigate whether AMH plays a role in the regulation of porcine luteal cell progesterone production.

In contrast to rodents where corpora lutea of four to five successive ovulation cycles can be found, corpora lutea in the pig ovary undergo luteolysis at the end of the luteal phase of the oestrus cycle when no fertilization of oocytes has occurred [34]. This means that at the beginning of the follicular phase of the oestrus cycle when the next cohort of small antral follicles is recruited to enter the growing phase, these AMH-positive corpora lutea have degenerated and therefore it is not likely that the growth of these small antral follicles will be influenced by luteal cell derived AMH.

The availability of sensitive AMH immunoassays has made it possible to determine AMH levels in multiple species. Recently, circulating AMH concentrations have been positively associated with the ovarian population of gonadotropin-responsive follicles in prepubertal hormone treated goats and cattle [47, 48]. It would be interesting to determine if this is applicable to the pig as well. However, because porcine corpora lutea also expresses AMH and thus most likely contribute to circulating AMH levels at least during the luteal phase of the cycle, it is advised when investigating whether AMH can be used as predictor of the gonadotropin-sensitive follicle pool size, to measure plasma AMH concentrations during the follicular phase of the oestrus cycle.

In conclusion, the results of the present study show that, like in the human ovary, AMH staining appears in theca cells of preovulatory follicles. However, in contrast to the human ovary and any other species reported so far, in the pig AMH persists after ovulation in the granulosa and theca luteal cells (Table 1, [49–69]). We hypothesize therefore, that the persistent presence of AMH in the corpora lutea after ovulation at this stage of the oestrous cycle may decrease the responsiveness of small antral follicles to FSH, thus contributing to the inhibition of premature recruitment and subsequent atresia of these small antral follicles.

## Supporting information

S1 Fig Representative AMH staining (brown) of the porcine ovary. (A) Small piece of the cortex, (B) corpus luteum. 1—quiescent primordial follicle; 2—recruited primordial follicle, 3—primary follicle, 4—preantral follicle, ST—stroma, CL—corpus luteum. Scale bar represents 60 μm (A) and 80 μm (B), respectively.
(PDF)

S2 Fig Representative AMH staining (brown) in the rat ovary. (A) granulosa cells of a primary follicle (arrowhead) and a small preantral follicle (arrow). (B) Early antral follicle. Scale bar represents 21 μm.
(PDF)
