# Supplementary figures and images for: Presence of anti-Müllerian hormone (AMH) during follicular development in the porcine ovary
Source: PLoS One. 2018 Jul 31;13(7):e0197894. doi: 10.1371/journal.pone.0197894 (PMC6067700; doi:10.1371/journal.pone.0197894)

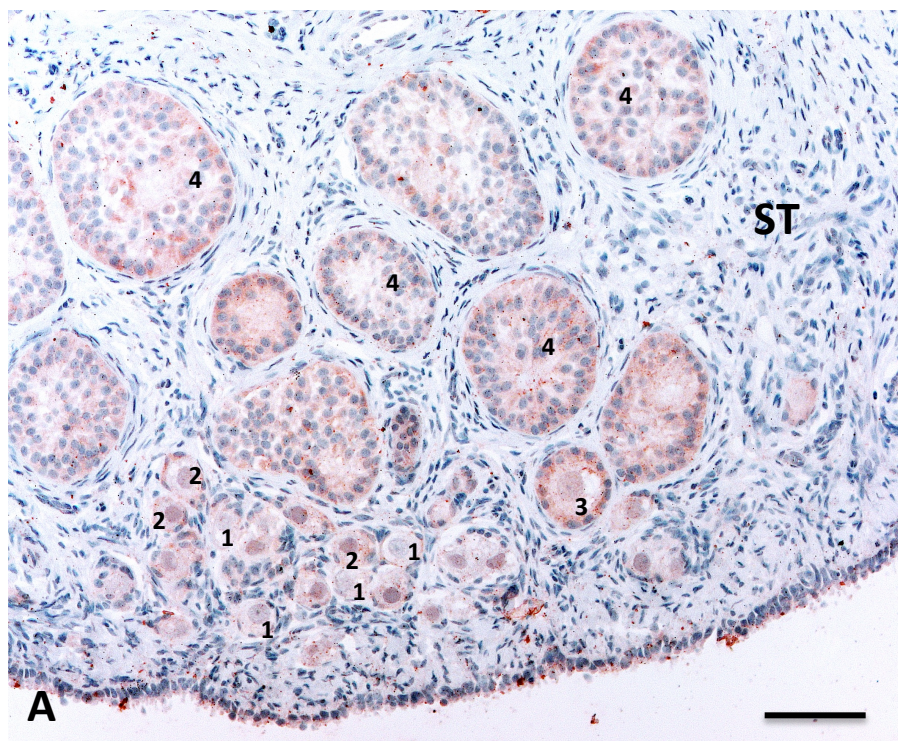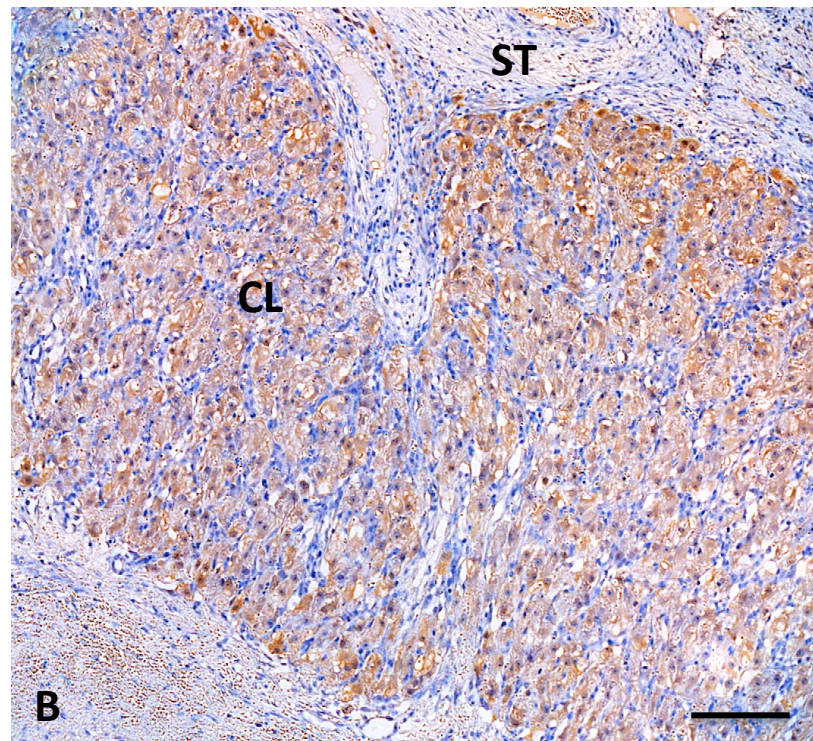

Supplemental figure 1

Supplement: S1 Fig — (A) Small piece of the cortex, (B) corpus luteum. 1—quiescent primordial follicle; 2—recruited primordial follicle, 3—primary follicle, 4—preantral follicle, ST—stroma, CL—corpus luteum. Scale bar represents 60 μm (A) and 80 μm (B), respectively. (PDF) [file pone.0197894.s001.pdf]

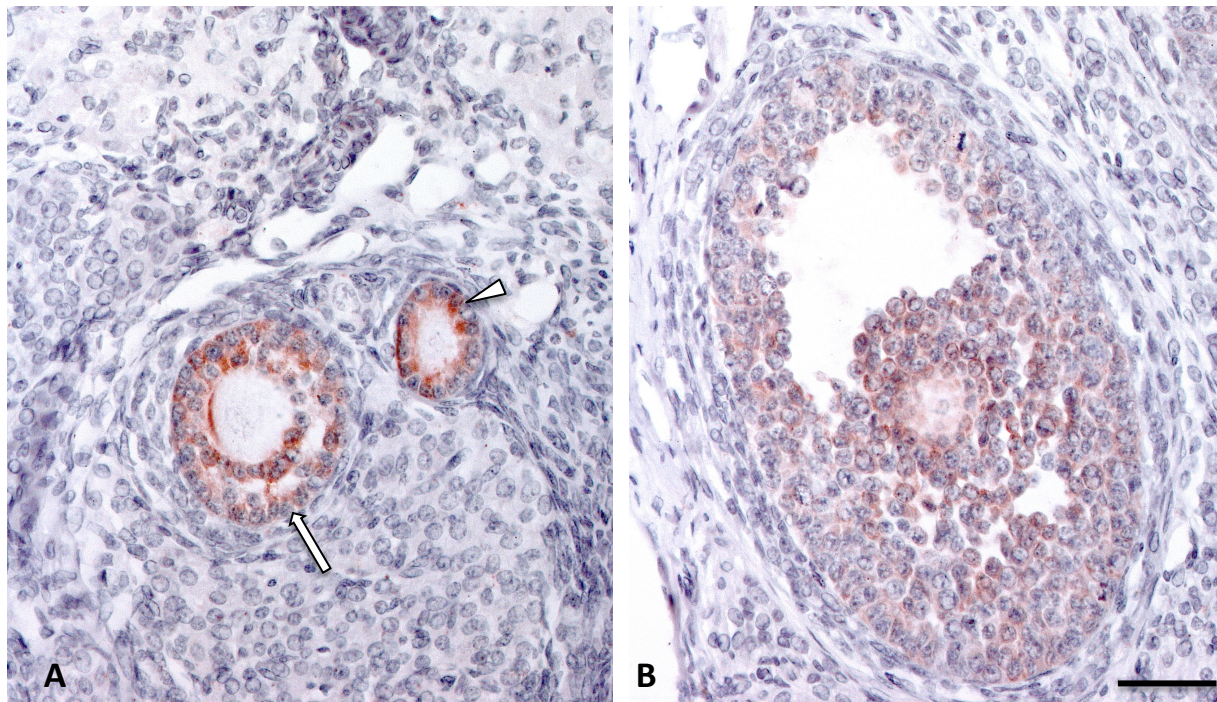

Supplemental figure 2

Supplement: S2 Fig — (A) granulosa cells of a primary follicle (arrowhead) and a small preantral follicle (arrow). (B) Early antral follicle. Scale bar represents 21 μm. (PDF) [file pone.0197894.s002.pdf]
